# Recognition and Activation Domains Contribute to Allele-Specific Responses of an Arabidopsis NLR Receptor to an Oomycete Effector Protein

**DOI:** 10.1371/journal.ppat.1004665

**Published:** 2015-02-11

**Authors:** Adam D. Steinbrenner, Sandra Goritschnig, Brian J. Staskawicz

**Affiliations:** Department of Plant and Microbial Biology, University of California, Berkeley, Berkeley, California, United States of America; CSIRO, AUSTRALIA

## Abstract

In plants, specific recognition of pathogen effector proteins by nucleotide-binding leucine-rich repeat (NLR) receptors leads to activation of immune responses. RPP1, an NLR from *Arabidopsis thaliana*, recognizes the effector ATR1, from the oomycete pathogen *Hyaloperonospora arabidopsidis*, by direct association via C-terminal leucine-rich repeats (LRRs). Two RPP1 alleles, RPP1-NdA and RPP1-WsB, have narrow and broad recognition spectra, respectively, with RPP1-NdA recognizing a subset of the ATR1 variants recognized by RPP1-WsB. In this work, we further characterized direct effector recognition through random mutagenesis of an unrecognized ATR1 allele, ATR1-Cala2, screening for gain-of-recognition phenotypes in a tobacco hypersensitive response assay. We identified ATR1 mutants that a) confirm surface-exposed residues contribute to recognition by RPP1, and b) are recognized by and activate the narrow-spectrum allele RPP1-NdA, but not RPP1-WsB, in co-immunoprecipitation and bacterial growth inhibition assays. Thus, RPP1 alleles have distinct recognition specificities, rather than simply different sensitivity to activation. Using chimeric RPP1 constructs, we showed that RPP1-NdA LRRs were sufficient for allele-specific recognition (association with ATR1), but insufficient for receptor activation in the form of HR. Additional inclusion of the RPP1-NdA ARC2 subdomain, from the central NB-ARC domain, was required for a full range of activation specificity. Thus, cooperation between recognition and activation domains seems to be essential for NLR function.

## Author Summary

Plants defend themselves against pathogens using specific multi-domain immune receptors, which are able to recognize secreted “effector” proteins from the pathogen, and thus activate an immune response. Variants of the Arabidopsis immune receptor RPP1 recognize different alleles of the oomycete effector ATR1 through direct association. RPP1 and ATR1 alleles from different ecotypes and strains show a spectrum of recognition phenotypes, reflecting coevolution by the plant and pathogen to evade and re-establish immunity. In this work, we identified mutations in an unrecognized ATR1 allele that lead to allele-specific recognition by RPP1. Using chimeric constructs of the immune receptor, in which domains were swapped between two alleles, we were able to determine domains contributing to allele-specific activation. Our data point to the involvement of two domains in specific activation of immune receptors—one to associate with the effector, and one to sensitize the receptor and facilitate activation. We suggest that these domains must cooperate to efficiently and specifically recognize pathogen effectors. As NLRs confer pathogen resistance in many crop species, characterizing specificity domains involved in effector recognition will inform future efforts to breed or engineer disease resistant varieties.

## Introduction

A critical step in the lifestyles of plant pathogens is the secretion of effectors—pathogen-encoded proteins that are translocated into the plant cell, where they manipulate the host and promote pathogen growth [1]. Many effectors function to modulate basal immunity, but their presence in the plant cell may betray the pathogen and activate a second layer of effector-triggered immunity (ETI) if recognized by intracellular host immune receptors [2–4]. Most effector recognition occurs via the NLR family of immune receptors (for nucleotide-binding and leucine-rich repeat protein). NLR activation results in an elevated immune response, characterized by generation of reactive oxygen species, activation of defense-associated genes, and a localized cell death known as the hypersensitive response (HR) [5].

Multiple modes of effector-triggered NLR activation have been described. Well-studied plant NLRs, such as RPS2 and RPM1 from Arabidopsis, recognize effectors indirectly [6–8]. These NLRs are activated not by association with effectors themselves, but instead by recognizing their biochemical effects in the plant cell, leading to models of NLR activation in which the receptors recognize perturbation of “guarded” host proteins. Guarded proteins can be true virulence targets or simply decoys inviting modification by the pathogen [9,10]. In contrast, NLRs including RPP1, L6, Pi-ta, and others interact directly with recognized effector alleles, suggesting a second model in which direct effector-NLR interaction is required for immune activation [11–15].

The modular domain architecture of NLRs allows for the integration of effector recognition and signaling activation by a switch-like mechanism [16]. An N-terminal domain, usually either a coiled-coil or Toll/interleukin-1-receptor (TIR) domain, mediates downstream immune signaling [17,18] and is followed by a nucleotide binding (NB) domain and two helical ARC subdomains (for Apaf-1, R protein, CED-4). This composite NB-ARC domain functions as a switch through exchange of an internally bound ADP for ATP [19,20], but may also participate in cis-regulatory interactions in order to maintain an ADP-bound “off” state [21–23]. These intramolecular interactions typically occur with a series of C-terminal leucine-rich repeats (LRRs), which are critical for auto-inhibition in the absence of effector [24,25]. A second role for the LRRs is in effector recognition, where the protein-protein interaction capacity of LRRs can mediate effector binding [12,13]. Consistent with this role, NLR recognition specificity can be expanded through LRR variation [26,27]. Positively selected amino acids in Arabidopsis NLR proteins genome-wide are also disproportionately located in the LRRs, consistent with LRR co-evolution with effector proteins under selective pressure to evade recognition [28].

Recognition of the oomycete effector ATR1 by the Arabidopsis NLR RPP1 is consistent with the direct interaction model of receptor activation, and serves as a model system for studying the molecular basis of NLR function [29]. ATR1 is one of approximately 140 effectors expressed and secreted by the naturally-occurring Arabidopsis pathogen *Hyaloperonospora arabidopsidis (Hpa)* [30], and is recognized specifically by the NLR protein RPP1, leading to *Hpa* resistance [31]. Diverse *ATR1* alleles from *Hpa* strains encode effectors that are differentially recognized by RPP1 [12,32], and thus *ATR1* can condition strain-dependent resistance on a given Arabidopsis ecotype [33]. Variation in the RPP1 receptor also contributes to the spectrum of resistance phenotypes; for example, RPP1-NdA (from the Niederzenz ecotype) and RPP1-WsB (from the Wassilewskija ecotype) vary in recognition specificity, with RPP1-NdA recognizing a smaller subset of the ATR1 alleles recognized by RPP1-WsB [12].

As *Hpa* is an obligate biotroph, surrogate systems are used to study the molecular basis for ATR1 recognition by RPP1. Alleles of ATR1 and RPP1 can be co-expressed in *Nicotiana tabacum*, resulting in a visible HR only for combinations in which RPP1 is able to recognize the ATR1 variant [12]. Biochemical and genetic lines of evidence from this system support a direct interaction model of ATR1 recognition. Co-immunoprecipitation of ATR1 alleles with RPP1-WsB correlates with HR activation capability [12], suggesting that direct interaction with the effector leads to receptor activation and signaling for ETI. Furthermore, the LRR domain of RPP1-WsB is sufficient for association with ATR1, indicating a role for the LRRs in effector recognition. ATR1 has no known virulence function, and adopts a WY-domain fold common to oomycete effector proteins [34], specifically with an N-terminal three-helix bundle and two tandem WY-domains comprising the “head” and “body” of a seahorse-like structure, respectively [35]. Single amino acid substitutions on both the head and body region can confer gain-of-recognition phenotypes to an unrecognized ATR1 allele, ATR1-Cala2 [35]. The surface-exposure of these substituted residues is consistent with these substitutions altering protein-protein interaction strength between ATR1 and RPP1 [12,35].

In this work, we address several outstanding questions regarding NLR receptor activation using the ATR1-RPP1 system. We performed a random mutagenesis screen of unrecognized ATR1-Cala2 to generate combinations of ATR1 mutations that activate each RPP1 allele. We show that different ATR1 mutants specifically associate with and activate resistance against either RPP1-NdA or RPP1-WsB, defining distinct recognition specificities for the two RPP1 alleles. We then used these ATR1 mutants to probe recognition and activation domains of both RPP1 alleles. Chimeric receptors revealed that while the LRRs were sufficient for recognition of ATR1 through molecular association, they were insufficient to recapitulate a receptor’s full range of specificity. Instead, inclusion of the ARC2 subdomain is further required for effective receptor activation.

## Results

### Mutagenesis screen reveals gain-of-function phenotypes in ATR1-Cala2

Previous work in our lab employed natural variation across ATR1 alleles to identify effector surfaces involved in recognition by RPP1. Amino acids conserved in recognized ATR1 variants were substituted into the distantly related and unrecognized allele, ATR1-Cala2. Four substitutions were each sufficient to give gain-of-recognition HR phenotypes by RPP1-WsB, but not RPP1-NdA, and combining all four substitutions led to a robust response with similar timing and intensity to the naturally recognized allele, ATR1-Emoy2 [35]. Here, we term this combined mutant ATR1-Cala2 WsB-GOF (Gain-of-Function). In combination with the crystal structure of ATR1, these results indicated that surface-exposed residues on a central WY-domain were involved in recognition by RPP1-WsB [35].

**Fig 1:**
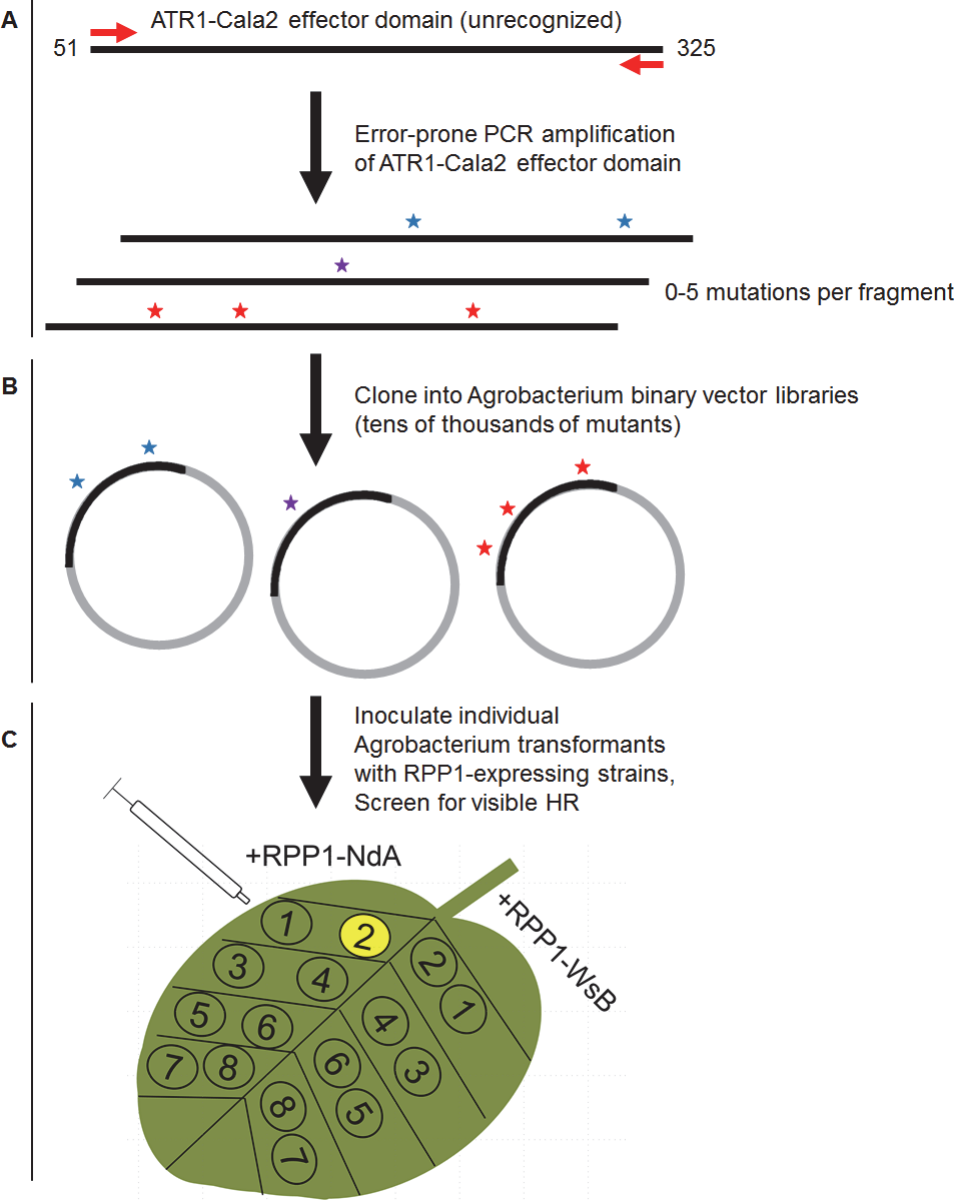
Overview of random mutagenesis screening process for gain-of-function ATR1-Cala2 mutations. (**A)** Error-prone PCR of the effector domain of ATR1 (a Δ51 N-terminal truncation) results in 0–5 mutations per construct. **(B)** LR recombination of PCR products into binary vector in frame with FLAG epitope tag, transformation of *E*. *coli*, and pooling of mutant clone populations. 6 separate libraries from independent PCR reactions yielded ∼10,000 distinct *E*. *coli* clones. **(C)** Binary vector preparations were transformed into *Agrobacterium* by electroporation and individual clones were co-expressed with RPP1-NdA or RPP1-WsB to test for hypersensitive response (HR) phenotypes.

**Fig 2:**
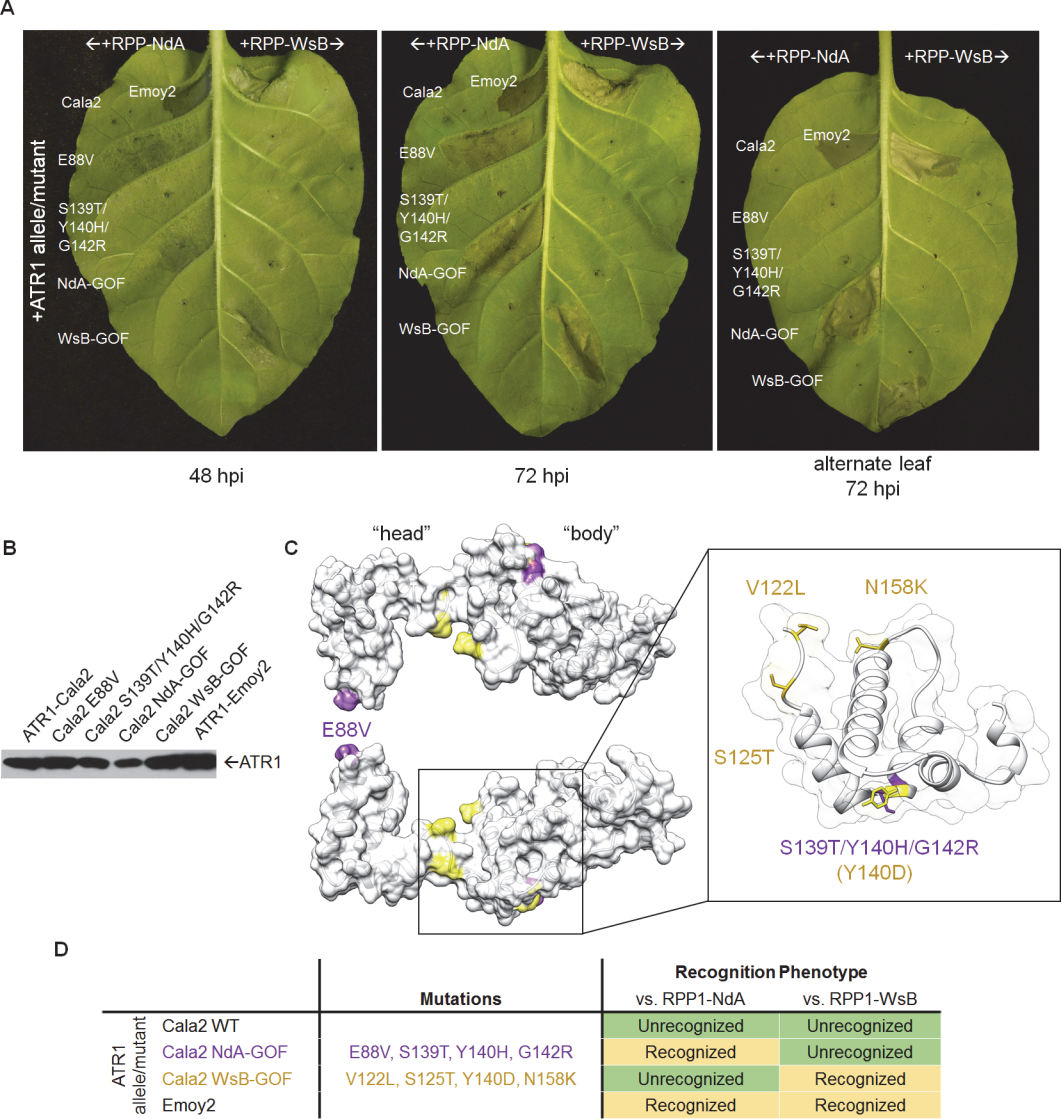
ATR1-Cala2 gain of recognition mutations yield RPP1-NdA specific hypersensitive response (HR). **(A)** Co-expression of ATR1-Cala2 mutants recovered from random mutagenesis screen with RPP1. Mutant ATR1-expressing *Agrobacterium* strains were inoculated with RPP1-NdA or RPP1-WsB-expressing strains at OD = 0.45 per construct, with unrecognized (ATR1-Cala2) and recognized (ATR1-Emoy2) alleles as controls. ATR1-Cala2 NdA-GOF comprises E88V/S139T/Y140H/G142R. HR cell death was photographed at 48 hour post inoculation (hpi) for weaker, incomplete response or 72 hpi for a complete response. **(B)** Western blot of FLAG-tagged ATR1-Cala2, Emoy2, and mutant alleles. Protein was collected at 24 hpi **(C)** Homology model of ATR1-Cala2 to the seahorse-like ATR1-Emoy2 crystal structure, with “head” and “body” positions of RPP1-NdA specifying mutations (NdA-GOF) in purple and previously identified RPP1-WsB specifying mutations (WsB-GOF) in yellow. An enlarged portion comprising the central “body” WY-motif is shown, with mutations highlighted as above. Y140 was mutated in both NdA- and WsB-GOF ATR1 mutants and is highlighted in yellow. **(D)** Summary of recognition phenotypes of ATR1 alleles and mutants against both alleles of RPP1.

While single amino acid substitutions can confer RPP1-WsB recognition, no natural alleles or mutants of ATR1 exclusively activate RPP1-NdA. To test whether other ATR1 surfaces could confer gain-of-recognition phenotypes, and whether allele-specific mutants could be obtained, we performed a random mutagenesis screen of ATR1-Cala2 for gain-of-recognition by either RPP1 allele (Fig. 1). We transiently co-expressed 2,240 clones of *Agrobacterium tumefaciens* expressing the mutagenized effector domain (Δ51) of ATR1-Cala2 with RPP1-NdA and RPP1-WsB in *Nicotiana tabacum* and screened for visible cell death HR, indicating NLR receptor activation. Only two mutants were recovered from the screen: either a valine substitution at position 88 (E88V) or a set of three combined substitutions at positions 139, 140, and 142 (S139T/Y140H/G142R) was each sufficient to confer weak recognition by RPP1-NdA, but not RPP1-WsB at 48hpi, and both mutants developed stronger activation of RPP1-NdA by 72hpi (Fig. 2A). Allele-specific responses could be combined, as combining E88V with the previously described WsB-GOF substitutions led to recognition by both RPP1 alleles (S1 Fig.). HR strength varies by leaf age, and older, less sensitive leaves did not show similar gain-of-recognition phenotypes for either mutant. However, combining all four substitutions into a single construct, termed ATR1-Cala2 NdA-GOF, allowed for more robust activation of RPP1-NdA than either individual substitution (Fig. 2A right, alternate leaf), albeit still weaker than activation by ATR1-Emoy2 or ATR1-Cala2 WsB-GOF of their respective RPP1 alleles (Fig. 2A left, 48 hpi). All ATR1 mutants expressed to a similar level to the unrecognized WT ATR1-Cala2 allele (Fig. 2B). Thus, mutant variants of ATR1-Cala2 activate RPP1-NdA but not RPP1-WsB, defining unique recognition specificities for each RPP1 allele.

### Multiple ATR1 surfaces condition RPP1-NdA recognition

We next mapped the NdA-GOF mutations onto a homology model of the ATR1-Cala2 structure, using the solved ATR1-Emoy2 structure as a template (Fig. 2C, see S2 Fig. for amino acid alignment). All four substitutions are predicted to be completely or partially surface exposed (>25 Å^2^ exposed), consistent with a predicted role in direct interaction with RPP1. All four substitutions also fall within the previously defined minimal region for RPP1 recognition [35], further indicating that this helical region specifies recognition by RPP1-NdA. E88V occurs on an N-terminal three-helix bundle. S139T/Y140H/G142R occur on the first of two tandem WY-domain repeats, at the N-terminal portion of the α1 helix as defined across other RXLR-type oomycete effectors [34,36]. Thus mutations on the specific surfaces on the “head” and “body” of the seahorse-like ATR1 structure can lead to specific activation of RPP1-NdA, but not RPP1-WsB, as summarized in Fig. 2D.

**Fig 3:**
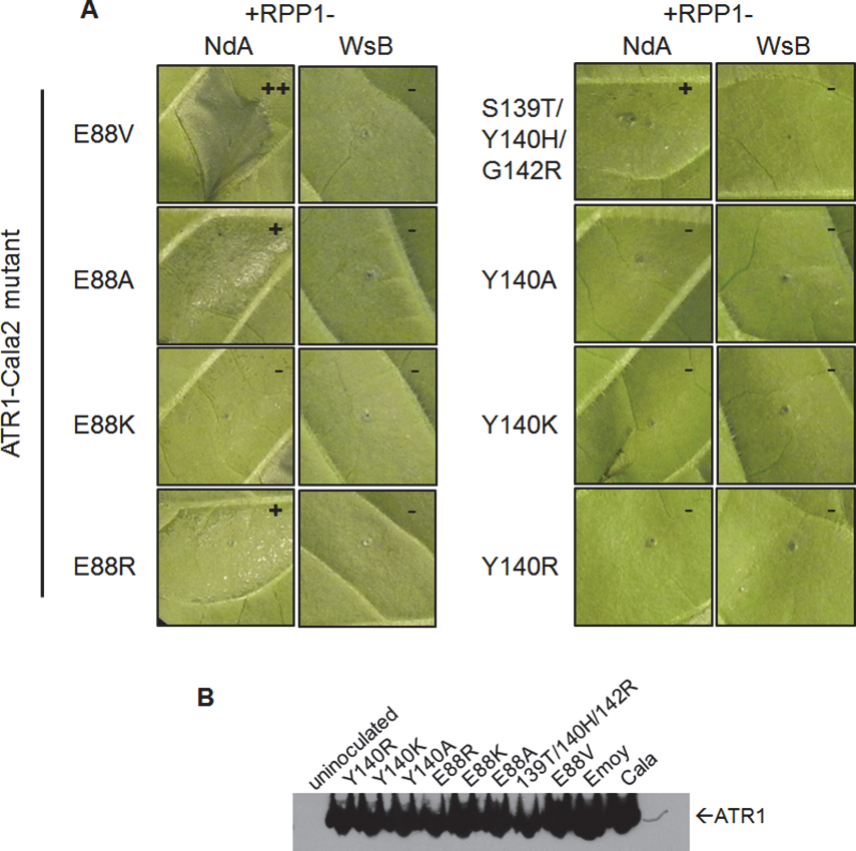
Charge-substituting mutations on ATR1 recognition surfaces variably alter allele-specific HR. **(A)** Co-inoculation of original ATR1-Cala2 Nda-GOF component substitutions (top row) and charge-altering variants. Constructs were co-inoculated as in Fig. 2A and photographed at 48 hpi. Relative strength of HR is indicated with symbols, +++, strong HR, +, weak HR, -, no HR. **(B)** Western blot of ATR1 mutants, as in Fig. 2B.

Three of the four constituent substitutions in the ATR1-Cala2 NdA-GOF mutant altered the predicted ATR1 surface charge. These three mutations either substitute a positively charged side chain for a neutral side chain (Y140H, G142R) in the first WY-domain or a neutral side chain for a negative side chain (E88V) in the N-terminal three-helix bundle. We hypothesized that any alteration in surface charge on these ATR1 surfaces would affect recognition by RPP1. This is further supported by the fact that one of the component NdA-GOF substitutions, Y140H, occurs at the same residue as a previously identified WsB-GOF substitution, Y140D [35]. We generated alanine, lysine, and arginine substitutions at positions 88 and 140 in an ATR1-Cala2 background. Only two of these substitutions, E88R and E88A, conferred weak gain-of-recognition by RPP1-NdA but not RPP1-WsB (Fig. 3A). This HR reaction was, however, weaker than the originally identified mutation, E88V, despite similar expression levels of all mutants (Fig. 3B). Thus, simple changes in surface charge do not provide a consistent pattern of recognition phenotypes against RPP1-NdA or RPP1-WsB.

### Association of ATR1 and RPP1 *in vivo* correlates with HR phenotype

**Fig 4:**
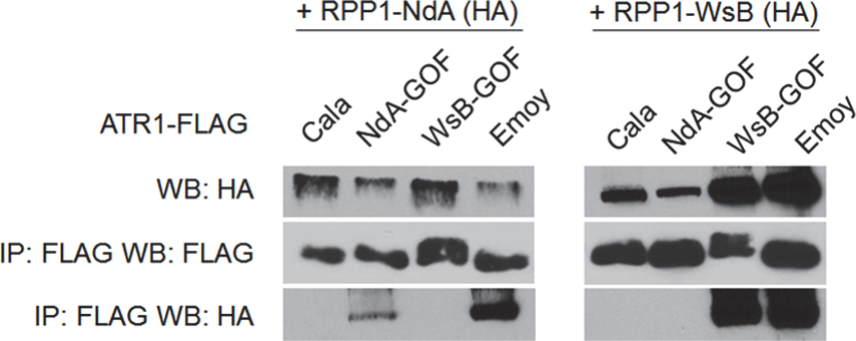
Exclusively recognized ATR1-Cala2-GOF mutants co-immunoprecipitate respective RPP1 alleles. FLAG-tagged ATR1 alleles were immunoprecipitated from *N*. *benthamiana* tissue extracts and probed for association with HA-tagged RPP1 by Western blot.

Previously, the ability of ATR1 alleles to co-immunoprecipitate RPP1-WsB correlated with activation of HR upon transient co-expression [12]. We tested whether the novel gain-of-recognition HR phenotypes of ATR1-Cala2 NdA-GOF and WsB-GOF also correlated with allele-specific RPP1 association. ATR1-Cala2 WsB-GOF associated with RPP1-WsB at a level similar to the recognized allele, ATR1-Emoy2, and did not associate with RPP1-NdA (Fig. 4). ATR1-Cala2 NdA-GOF did not associate with RPP1-WsB, but associated with RPP1-NdA, although more weakly than ATR1-Emoy2 (Fig. 4), consistent with the weaker HR phenotype we observed for this mutant (Fig. 2A). Overall, the association of ATR1 mutants with RPP1 correlates with HR phenotypes in tobacco, consistent with direct interaction of these mutants with corresponding RPP1 proteins.

### Type-three delivery of ATR1 GOF mutants leads to allele-specific RPP1-mediated resistance

Although *Hpa* is an obligate biotroph and has not been successfully cultured or genetically manipulated [32], surrogate approaches allow delivery of *Hpa* effectors into the Arabidopsis host by bacterial type three delivery to assay for induced host defense responses [37,38]. We tested whether HR and association phenotypes for ATR1 GOF mutants observed in transient assays correlated with resistance phenotypes in Arabidopsis upon bacterial delivery. ATR1 alleles and mutants were fused with the secretion signal of AvrRps4, mediating delivery by the endogenous type-three secretion system (TTSS) of strain DC3000 of the virulent bacterium, *Pseudomonas syringae* pv. *tomato*. Strains delivering alleles and mutants of ATR1 were inoculated into the recombinant inbred Arabidopsis line HRI3860, which lacks functional *RPP1* [32], as well as transgenic HRI3860 lines expressing RPP1-NdA or RPP1-WsB.

**Fig 5:**
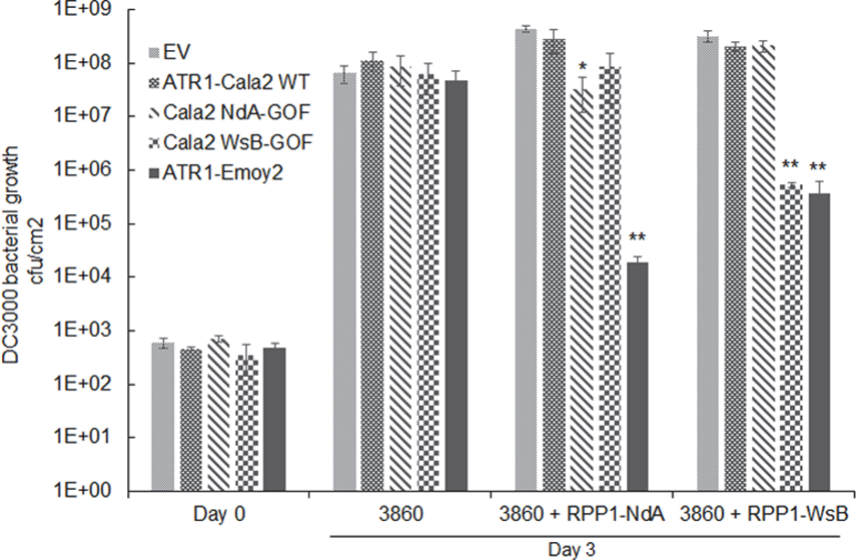
Delivery of alleles and mutants of ATR1 by *Pseudomonas syringae* pv. *tomato* strain DC3000 leads to allele specific growth inhibition. Plant lines were inoculated with pEDV3 constructs containing ATR1 variants and inoculated at OD = 0.0001. *, p<0.05, **, p<0.01, Student’s T-test.

While all strains grew to similar levels by 3 days post-inoculation (dpi) on HRI3860 plants, delivery of ATR1-Emoy2 strongly inhibited bacterial growth in transgenic lines expressing either RPP1 allele (Fig. 5A). Strains with either an empty vector or delivering ATR1-Cala2 were uninhibited in growth on either transgenic line. ATR1-Cala2 WsB-GOF delivery strongly inhibited bacterial growth on the RPP1-WsB expressing line, while ATR1-Cala2 NdA-GOF weakly inhibited growth on the RPP1-NdA expressing line (Fig. 5A). Disease symptoms at 3 dpi correlated with growth inhibition. While ATR1-Cala2 delivering strains remained visibly virulent on both transgenic lines, producing chlorosis and necrotic lesions, delivery of ATR1 GOF alleles led to RPP1-NdA or RPP1-WsB-specific avirulence, leading to a healthy phenotype similar to that observed after delivery of the fully recognized allele, ATR1-Emoy2 (S3A Fig.).

We also tested whether gain-of-recognition alleles could elicit HR phenotypes in the same RPP1-expressing transgenic lines. Delivery of different ATR1 alleles by *Pseudomonas fluorescens* (*Pf0*) engineered to express a TTSS was previously shown to yield allele-specific HR in Arabidopsis, correlating with HR phenotypes in the transient tobacco assay [33,39]. The ATR1-Cala2 WsB-GOF mutant elicited a visible HR on a RPP1-WsB transgenic line, but we were unable to detect activation of strong HR by *Pf0* delivering ATR1-Cala2 NdA-GOF to an RPP1-NdA transgenic line (S3B Fig.). This weaker HR recognition phenotype of the NdA-GOF mutant is comparable to growth inhibition, Co-IP, and transient HR phenotypes (Fig. 2A, Fig. 4, Fig. 5A).

### Chimeric RPP1 proteins indicate a specificity role for the RPP1-NdA ARC2 subdomain

**Fig 6:**
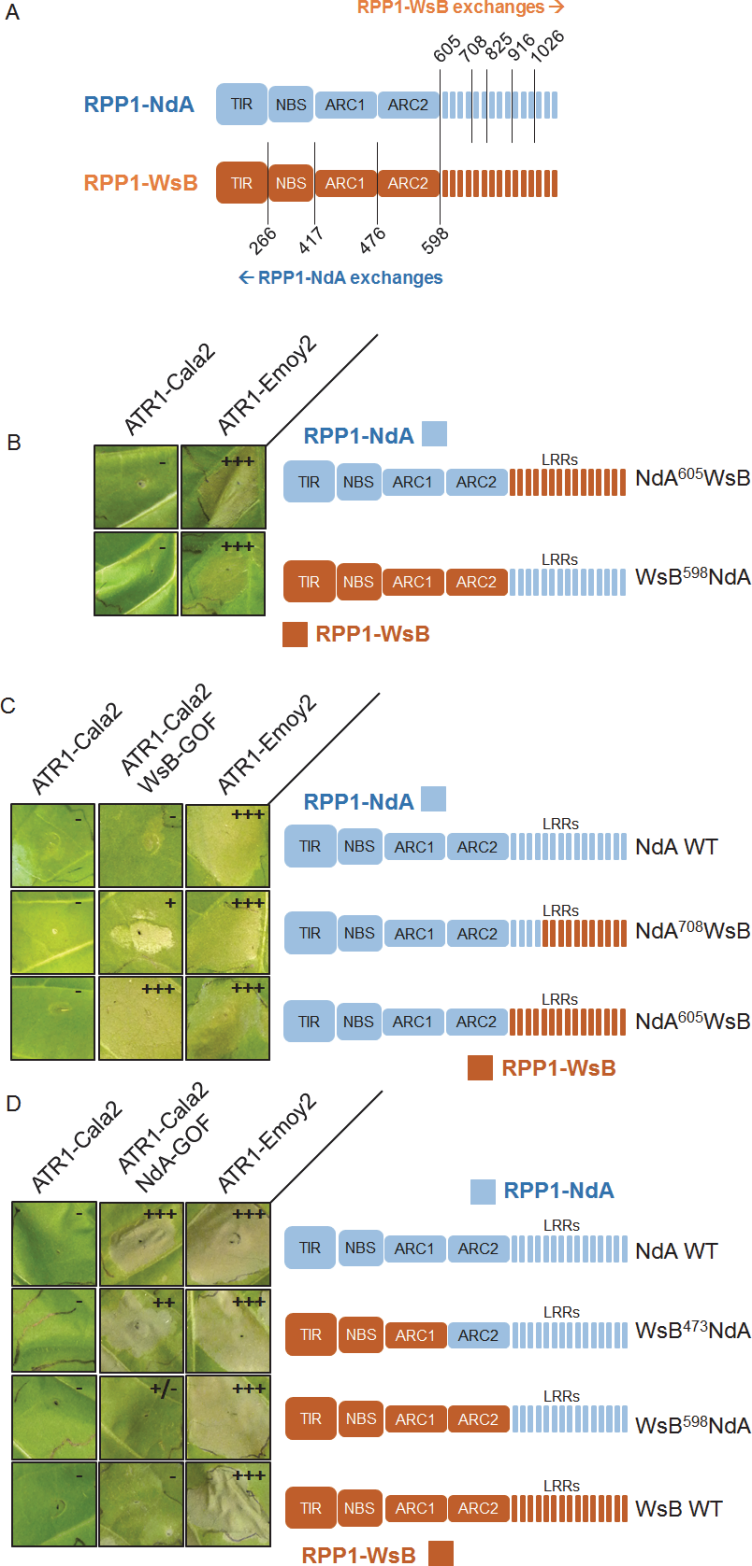
Chimeric RPP1 constructs implicate LRRs and ARC2 domains in specificity. **(A)** Amino acid numbering for chimeric exchanges. Colors (light blue for RPP1-NdA, dark orange for RPP1-WsB) indicate domains with either RPP1-NdA or RPP1-WsB amino acid identity. See S4 Fig. for chimeric constructs in context of pairwise sequence alignment. **(B)** Chimeric constructs of RPP1-NdA and RPP1-WsB with complete LRR exchanges tested against ATR1 alleles with conserved recognition. **(C)** N-terminal LRRs mediate recognition of ATR1-Cala2 WsB-GOF. Tobacco HR phenotypes against ATR1-WsB-GOF are shown, with additional chimeric exchanges within LRRs. **(D)** RPP1-NdA ARC2 domain is required for activation by ATR1-Cala2 NdA GOF. Tobacco HR phenotypes against ATR1-Cala2 NdA-GOF are shown, with additional chimeric exchanges N-terminal to the LRRs. See S5 Fig. for additional non-functional chimeric constructs and visible mixed (+/-) recognition phenotype by WsB^598^NdA. Inoculations were performed as in Fig. 2A, with RPP1 construct indicated on the right. Relative strength of HR is indicated with symbols, +++, strong HR, ++, moderate HR, +, weak HR, +/-, mixed HR, -, no HR.

Despite the weak recognition and resistance phenotypes activated by ATR1-Cala2 NdA-GOF relative to those of ATR1-Cala2 WsB-GOF, the exclusivity of the mutants for activating either RPP1-NdA or RPP1-WsB allowed us to explore regions governing recognition and activation for each RPP1 protein. We hypothesized that, since NdA-GOF and WsB-GOF mutations occurred on different ATR1 surfaces (Fig. 2C), unique receptor regions might be responsible for recognition of each mutant. We generated several chimeric RPP1 constructs to test the contribution of different NLR domains to activation (Fig. 6A). As the leucine-rich repeats (LRRs) of RPP1-WsB are sufficient for association with ATR1-Emoy2 [12], we first generated reciprocal chimeric constructs between RPP1-NdA and RPP1-WsB in which all predicted C-terminal LRRs were swapped (see S4 Fig. for chimeric exchange points on pairwise sequence alignment). Neither WsB LRRs in an NdA context (NdA^605^WsB) nor NdA LRRs in a WsB context (WsB^598^NdA) led to autoactivity or activation by ATR1-Cala2, and both retained the conserved ability to recognize ATR1-Emoy2 (Fig. 6B).

We next tested whether the LRRs from one RPP1 allele were sufficient to recognize the cognate ATR1-Cala2 NdA-GOF or WsB-GOF mutant. As expected, NdA^605^WsB, a chimera with RPP1-WsB LRRs, was fully activated by ATR1-Cala2 WsB-GOF (Fig. 6C). To determine a potential cutoff point for recognition along the series of C-terminal LRRs, we constructed a series of LRR chimeras in which the chimeric exchange point was made every 4–5 repeats further C-terminal, based on homology-modeled LRRs [29]. Most chimeric exchanges were completely inactivated: NdA^825^WsB and NdA^916^WsB were detected by Western blot, but, unlike WT RPP1-NdA and RPP1-WsB, did not recognize ATR1-Emoy2 (S5A Fig.). A third chimeric LRR construct, NdA^1026^WsB, was not expressed (S5C Fig.). One chimera, however, NdA^708^WsB, expressed (S5C Fig.), recognized ATR1-Emoy2, but showed diminished HR in response to ATR1-Cala2 WsB-GOF relative to NdA^605^WsB (Fig. 6C), indicating that the first 4 LRRs of RPP1-WsB are required for full recognition of this mutant.

Surprisingly, a reciprocal chimera with RPP1-NdA LRRs, WsB^598^NdA, was only weakly activated by ATR1-Cala2 NdA-GOF (Fig. 6D), only visible as mild HR on the backside of the leaf (S5B Fig.). This indicated that the RPP1-NdA LRRs are insufficient for recapitulating the full range of RPP1-NdA specificity. We generated constructs with chimeric fusions further N-terminal relative to the LRRs, at the TIR-NBS, NBS-ARC1, and ARC1-ARC2 domain junctions. While a chimera exchanging the TIR domain (WsB^266^NdA) expressed but was inactive even in recognizing the conserved recognized ATR1 allele, ATR1-Emoy2 (S5A Fig.), all other constructs recognized ATR1-Emoy2 with similar timing and intensity (Fig. 6D, S4B Fig.). Chimeras with RPP1-NdA C-termini comprising either the ARC2-LRR (WsB^473^NdA) or ARC1-ARC2-LRR (WsB^417^NdA) domains were able to activate in response to ATR1-Cala2 NdA-GOF (Fig. 6D, S5B Fig.), with the strongest response generated by RPP1-NdA ARC2-LRRs in an RPP1-WsB context (WsB^473^NdA). Thus, while the RPP1-NdA LRRs are insufficient to allow activation by ATR1-Cala2 NdA-GOF, inclusion of an RPP1-NdA ARC2 domain in chimeric constructs allows for receptor activation and contributes to RPP1-NdA specificity.

### RPP1-NdA ARC2 subdomain is required for allele-specific activation, but not ATR1 recognition

**Fig 7:**
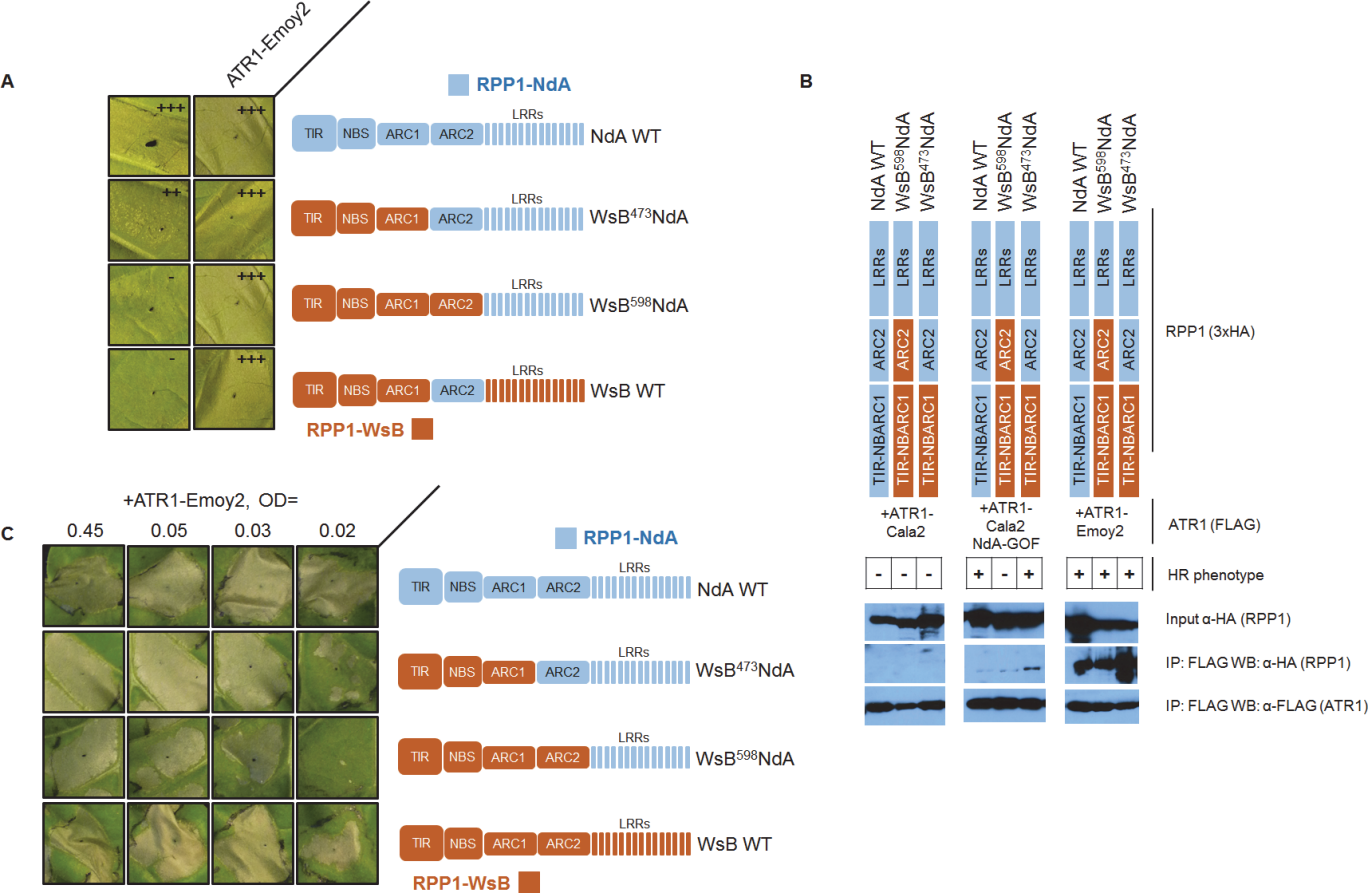
The RPP1-NdA ARC2 subdomain contributes to specificity by facilitating receptor activation. **(A)** RPP1-NdA ARC2 is insufficient for allele-specific recognition. Chimeric constructs, including a double chimera with RPP1-NdA ARC2 in a RPP1-WsB context were tested against NdA-specific (ATR1-Cala2 NdA-GOF) and conserved recognized (ATR1-Emoy2) ATR1 alleles. **(B)** RPP1-NdA ARC2 is dispensable for allele-specific recognition through association. FLAG-tagged ATR1 alleles were immunoprecipitated from *N*. *benthamiana* tissue extracts and probed for association with HA-tagged RPP1 by Western blot. HR phenotype is indicated with + or - in table. **(C)** RPP1-NdA ARC2 domain sensitizes the receptor to low inoculum of ATR1-Emoy2. Density of inoculum of Agrobacterium strain for ATR1-Emoy2 expression is indicated at top. Inoculations were performed as in Fig. 2A, with RPP1 construct indicated on the right. Relative strength of HR is indicated with symbols, +++, strong HR, ++, moderate HR, -, no HR.

The RPP1-NdA ARC2 domain could expand specificity in two possible ways—either the ARC2 domain is involved in recognition of the ATR1 mutant through allele-specific contacts, or it instead plays a role in facilitating activation upon ATR1 recognition by the LRRs. We carried out experiments to distinguish between these binding versus activation roles. A binding role would be supported by sufficiency of the RPP1-NdA ARC2 subdomain for both association and activation by ATR1-Cala2 NdA-GOF. To test an association role, we expressed only the NB-ARC or ARC1-ARC2 domains (amino acids 296–605 or 424–605 respectively) and probed for co-immunoprecipitation with ATR1-Cala2 NdA-GOF or ATR1-Emoy2. Co-immunoprecipitation of either domain with ATR1 was not observed (S6 Fig.), but the RPP1-NdA LRRs were also unable to interact with ATR1 in these experiments (S7 Fig.). We thus directly tested ARC2 sufficiency for activation by generating a double chimeric construct, WsB^473^NdA^605^WsB, with an RPP1-NdA ARC2 subdomain in an RPP1-WsB context (see cutoffs in S4 Fig.). This chimera did not activate in response to ATR1-Cala2 NdA-GOF (Fig. 7A). Thus the RPP1-NdA ARC2 domain is insufficient for activation by ATR1-Cala2 NdA-GOF, a finding inconsistent with a model where direct ARC2 contacts fully condition receptor activation.

An alternative, activation state model for expansion of specificity by the ARC2 domain is that the RPP1-NdA LRRs determine allele-specific recognition of ATR1, but a corresponding RPP1-NdA ARC2 domain is required for its full activation capacity. One prediction of this model is that RPP1-NdA LRRs should be sufficient to associate with the ATR1-Cala2 NdA-GOF mutant. Unlike with RPP1-WsB LRRs, we were unable to co-immunoprecipitate RPP1-NdA LRRs (amino acids 606–1164) even with the fully recognized allele, ATR1-Emoy2 (S7 Fig.). We were, however, able to investigate an LRR binding role by co-immunoprecipitating full-length chimeric constructs. While WT ATR1-Cala2 did not co-immunoprecipitate any chimeric RPP1 constructs, the ATR1-Cala2 NdA-GOF mutant associated with RPP1 chimeras containing an RPP1-NdA LRR, despite different activation phenotypes in the HR assay (Fig. 7B). Although this interaction of WsB^598^NdA with ATR1-Cala2 NdA-GOF was weaker than with ATR1-Emoy2, the level was similar to that of interaction with WT RPP1-NdA, suggesting that different receptor sensitivities, and not association strengths, condition the intensity of allele-specific HR.

Finally, the activation state model predicts that the threshold for specificity by a strongly recognized ATR1 allele will be lower for the desensitized WsB^598^NdA chimera. We tested transient expression of wild-type and chimeric RPP1 constructs against a gradient inoculum of a second Agrobacterium strain expressing ATR1-Emoy2. From typical to low inoculum (OD = 0.45 to OD = 0.03), ATR1-Emoy2 activates all constructs evenly (Fig. 7C). However, lowering the inoculum to OD = 0.02 led to specific activation of an RPP1-NdA ARC2-LRR containing construct, WsB^473^NdA, but not an LRR-only construct, WsB^598^NdA, consistent with a sensitizing effect of an allelic RPP1-NdA ARC2 domain. In summary, RPP1-NdA LRRs are sufficient for association with, but insufficient for full-strength activation by, an NdA-specific ATR1 mutant. Thus RPP1-NdA LRRs condition recognition, but efficient receptor activation further requires an RPP1-NdA ARC2 activation domain.

## Discussion

Allele-specific recognition of the *Hpa* effector ATR1 by the Arabidopsis RPP1 resistance protein serves as a model for activation of NLR immune receptors. In this work, we further characterized the ATR1-RPP1 interaction using gain-of-function ATR1 mutants in combination with a series of chimeric RPP1 constructs. Using novel allele-specific gain-of-function ATR1 mutants, we demonstrate that 1) two closely related NLR variants, RPP1-NdA and RPP1-WsB, have unique ATR1 recognition specificities, 2) substitutions conferring recognition by RPP1-NdA occur on multiple effector surfaces, and 3) the helical ARC2 domain of RPP1-NdA, in addition to the LRRs, functions as a specificity domain by facilitating receptor activation.

### Specific activation of RPP1-NdA or RPP1-WsB involves multiple ATR1 contacts

A set of mutations arising from our random mutagenesis screen of unrecognized ATR1-Cala2 conferred exclusive recognition by RPP1-NdA, but not RPP1-WsB (Fig. 2A). These NdA-GOF mutations occurred on both the “head” region of ATR1 (N-terminal three-helix bundle) and on the “body” (in the first of two tandem helical WY domains) (Fig. 2B). This structural location is consistent with previously described mutations of a differentially recognized allele, ATR1-Maks9, which is recognized by RPP1-WsB but not RPP1-NdA. Both a “head” substitution, E92K, and a “body” substitution, D191G, conferred RPP1-NdA recognition to ATR1-Maks9 [12]. Site-directed substitution of positively charged and neutral sidechains on these surfaces in ATR1-Cala2 did not lead to consistent gain-of-recognition phenotypes (Fig. 3), suggesting that more intricately defined surface interactions mediate specificity for each allelic pair. Nonetheless, the location of ATR1-Cala2 NdA-GOF substitutions is consistent with multiple surfaces contacting RPP1-NdA. The GOF residues described here and previously [12,35] also have a higher degree of surface exposure than the overall molecule (average of 81 Å^2^ relative to 65 Å^2^ for ATR1 overall), further consistent with surface contacts with RPP1. Molecule-level resolution data on these interacting surfaces, for example from crosslinking or crystallography experiments, will likely inform the basis of the recognition phenotypes described here.

The ATR1-Cala2 NdA-GOF mutant provides further evidence that direct activation of NLRs can show a gradient of response strength that depends on a variety of effector-NLR contacts. First, the four ATR1-Cala2 NdA-GOF substitutions are additive in strength of transient HR (Fig. 2A), consistent with multiple contact points with RPP1-NdA that quantitatively increase binding strength. The ATR1 Cala2 NdA-GOF activated responses were also weaker compared to that activated by the fully recognized allele, ATR1-Emoy2, in HR, co-immunoprecipitation, and bacterial growth inhibition assays (Fig. 2A, Fig. 4, Fig. 5A). Second, three of four residues substituted in ATR1-Cala2 NdA-GOF are also conserved in the recognized allele, ATR1-Emoy2 (S4 Fig.). This suggests that RPP1-NdA recognition of the ATR1-Emoy2 allele occurs via interaction with different amino acid residues. Together, these data indicate that RPP1-activated resistance is quantitative in strength and that different allelic combinations can depend on distinct ATR1-RPP1 contact points. This flexibility of the interaction likely contributes to high levels of amino acid variation in both the recognition domain of ATR1 and the LRR region of RPP1 [29]. Quantitative NLR activation strength may also underlie the partial resistance phenotypes observed for many *Hpa*-Arabidopsis interactions [33]. Formally, co-immunoprecipitation could indicate complex formation through an intermediate host factor. However, the nature of all gain-of-function ATR1 mutations described—surface substitutions activating specific RPP1 alleles in an additive fashion—is more consistent with recognition conditioned by direct association. Biochemical characterization of interaction strength between RPP1 and various ATR1 mutants may correlate affinity with subtle HR phenotypes described here, but these experiments await reliable methods for RPP1 protein expression and purification, which have been recalcitrant to date.

The ATR1-Cala2 NdA-GOF mutant also provides evidence for a high degree of specificity in the recognition spectra of RPP1 alleles. RPP1-NdA only recognizes a subset of naturally occurring ATR1 alleles recognized by RPP1-WsB [12,33], and thus prior to this study we could not distinguish between two competing hypotheses—either both alleles have distinct but overlapping recognition spectra, or RPP1-WsB is simply more sensitive to activation by a wider array of ATR1 variants. Support for the former, specificity-based model comes from our random mutagenesis screen. First, we did not recover any ATR1 mutants that activated RPP1-WsB; a more sensitive receptor might be expected to recognize a larger range of random mutants. Second, the fact that the mutant described here, ATR1-Cala2 NdA-GOF, is recognized by and associates with RPP1-NdA but not RPP1-WsB disproves the hypothesis that the expanded RPP1-WsB recognition spectrum is due to increased sensitivity. Rather, it is consistent with the closely related receptors having sophisticated, individual recognition abilities, in addition to their shared ability to recognize certain ATR1 variants. “Arms race” co-evolution of effector and receptor [40,41] likely leads to the unique specificities of the two RPP1 alleles described here. Pathogen-driven receptor diversity may also lead to autoimmune consequences for the host, as RPP1 variants from other Arabidopsis ecotypes can condition hybrid incompatibility through genetic interaction with other loci [42].

### The ARC2 domain of RPP1-NdA functions as a specificity domain by allowing more robust activation by ATR1 mutants

Allele-specific activation by the ATR1-Cala2 NdA-GOF and WsB-GOF mutants allowed us to explore recognition regions in the respective recognizing RPP1 alleles. A chimeric exchange placing RPP1-WsB LRRs in a RPP1-NdA context led to full ATR1-Cala2 WsB-GOF recognition (Fig. 6C), consistent with a recognition role for the LRRs. A smaller exchange excluding the first 4 LRRs gave a highly reduced recognition phenotype (Fig. 6C, NdA^605^WsB vs. NdA^708^WsB), while still fully recognizing ATR1-Emoy2. Polymorphic residues between RPP1-NdA and RPP1-WsB in these 4 LRRs likely mediate specificity for recognition of ATR1-Cala2 WsB-GOF (S8 Fig., left), including several residues on a predicted concave β-sheet surface associated with ligand binding in other LRRs [43].

Surprisingly, chimeric exchanges indicated a role of the RPP1-NdA ARC2 helical domain in activation. LRRs from RPP1-NdA did not allow for complete activation by ATR1-Cala2 NdA-GOF (Fig. 6D, WsB^598^NdA), but a further N-terminal chimeric exchange including the RPP1-NdA ARC2 domain (WsB^473^NdA) greatly strengthened the response. Further experiments testing chimeric, full-length RPP1 constructs against this ATR1 mutant indicated that the ARC2 domain expands specificity by facilitating receptor activation rather than by associating with the effector. The RPP1-NdA ARC2 domain in an RPP1-WsB context was insufficient for receptor activation by ATR1-Cala2 NdA-GOF, and an RPP1-NdA ARC2 domain was not required for its allele-specific ATR1 association (Fig. 7A,B). In addition, a chimera with RPP1-NdA ARC2 was able to activate in response to a lower inoculum of the fully recognized ATR1-Emoy2 allele (Fig. 7C). Thus the ARC2 domain of RPP1-NdA functions to expand its specificity by decreasing the threshold for activation of the receptor. We speculate that polymorphisms between RPP1-NdA and RPP1-WsB in the ARC2 domain condition intramolecular allelic compatibility between domains of the receptor, possibly through interactions with the LRRs. Several ARC2 polymorphisms occur on the predicted surface of a homology modeled ARC2, and could be candidates for LRR interaction (S8 Fig., right).

The structural basis of plant NLR receptor activation remains unclear, but there is increasing evidence for a role of the NB-ARC domain not just as a nucleotide hydrolysis-based switch downstream of effector perception [19], but as an active contributor to effector-triggered activation in combination with C-terminal LRRs. For example, recent data from chimeric exchanges in animal intracellular NLRs, the mouse NAIPs (NLR family, apoptosis inhibitory protein), corroborate a role for central helical domains in specificity. NAIPs oligomerize and recruit Caspase-1 upon recognition of specific ligands [44], and a series of chimeric exchanges between NAIP2 and NAIP5 indicated that specificity of oligomerization was determined by the second and third ARC-like helical domains rather than by the C-terminal LRRs [45]. Examples from plant NLRs also support a role for ARC domains in specificity. ARC helical domains of the flax receptor L can, in tandem with LRR polymorphisms, expand recognition of AvrL567 effector alleles [46]. Recently, ARC2 domain mutations were described in the wheat NLR Pm3 that expand its specificity against previously unrecognized wheat powdery mildew strains [47]. In contrast to inactivating or autoactivating mutations in other NLRs that map near the nucleotide binding pocket [24,48,49], the Pm3 recognition-expanding mutations map to a region of the ARC2 subdomain predicted to be an exposed loop [47], suggesting that the mutations affect intra- or intermolecular interactions. Intramolecular contacts between the ARC2 and LRR domains are thought to maintain an “off” state [23,25,46,50], and specific ARC2 amino acid substitutions can affect ARC2-LRR binding affinity [22]. Here we provide data that the ARC2 subdomain is dispensable for effector association, but required for full-strength activation, in an allele-specific effector-NLR interaction. Thus compatibility between ARC2 and LRRs, even in closely related NLR variants such as RPP1-NdA and RPP1-WsB, may be required for a full range of specificity by allowing efficient receptor activation.

**Fig 8:**
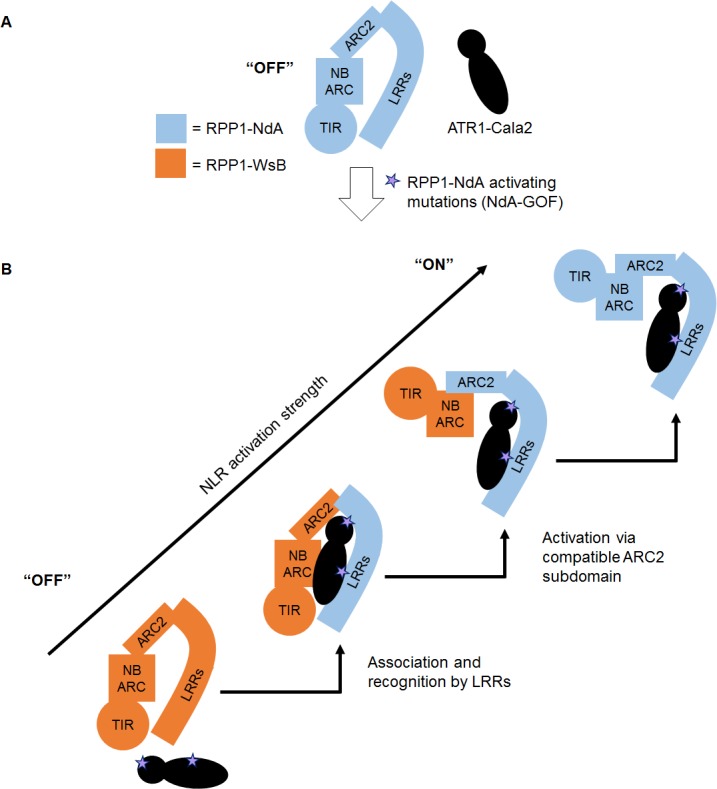
Model for RPP1 specificity involving recognition and activation domains. **(A)** Wild-type ATR1-Cala2 is unrecognized by RPP1, maintaining an “off” state conformation, potentially mediated by intramolecular ARC2-LRR interactions. **(B)** Specific amino acid substitutions in ATR1-Cala2 confer allele-specific recognition by RPP1-NdA (light blue). RPP1-NdA LRRs are sufficient for association with ATR1, but not for full-strength activation of the receptor. Further activation specificity is conferred by inclusion of an RPP1-NdA ARC2 domain. Full receptor activation strength occurs with wild-type RPP1-NdA, with full intramolecular compatibility.

We present a model of RPP1 function where cooperation between recognition by LRRs and activation by the ARC2 subdomain leads to a full-strength receptor response (Fig. 8). In an “off” state, RPP1-NdA is not activated by the unrecognized ATR1-Cala2 effector protein (Fig. 8A). Substitutions on distributed surfaces of ATR1-Cala2 allow activation of RPP1-NdA but not RPP1-WsB (Fig. 8B). Specificity is a multi-stage process: stepwise increases in recognition and activation strength in response to the ATR1 mutant can be achieved by substituting the recognition domain (LRRs) and activation domain (ARC2) from RPP1-NdA. Complete activation strength is achieved with full intramolecular compatibility in the wild-type RPP1-NdA receptor. While our data provide new insight into the roles for specificity domains of ATR1 and RPP1, it remains to be seen precisely how molecular contacts between the effector and receptor relieve plant NLR autoinhibition.

## Materials and Methods

### Bacterial growth conditions and constructs

*Escherichia coli* strain DH5α was used for cloning and propagation of pEarleyGate and pEDV3 constructs, and was grown at 37°C in LB or LB agar supplemented with 25 μg /mL kanamycin or 10μg/mL gentamycin. Agrobacterium strain GV3101::pMP90 [51] was propagated at 28°C in LB supplemented with 50 μg/mL gentamycin. *Pseudomonas* strains were propagated at 28°C. *Pseudomonas fluorescens* (*Pf0*) was grown on *Pseudomonas* Agar solid medium supplemented with 50μg/mL tetracyclin, 30 μg/mL chloramphenicol and 50 μg/mL gentamycin and *Pseudomonas syringae* pv. *tomato* DC3000 (*Pst* DC3000) was grown on NYGA solid medium supplemented with 100 μg/mL rifampicin and 5 μg/mL gentamycin.

FLAG-tagged ATR1 constructs were generated by attaching a primer encoded linker on the reverse primer, amplifying by PCR, and cloning into pENTR/D-TOPO (Invitrogen).

### Mutagenesis and tobacco transient HR assays

Error-prone PCR was carried out using the Diversify Random Mutagenesis Kit (Clontech) using condition 5 (640 μM manganese sulfate) to amplify the Δ51 ATR1-Cala2-FLAG coding sequence. PCR reactions were recombined into pEarleyGate 202 [52] by LR recombination (Invitrogen) and populations of bacterial clones were pooled into libraries of random mutants. Prepared pEG202 ATR1-Cala2 from these libraries was transformed into *Agrobacterium tumefaciens* strain GV3101 by electroporation, and individual clones were selected for co-inoculation. Site-directed mutations were introduced into pENTR/D-TOPO ATR1-Cala2 through mutagenic primers using the QuikChange XL kit (Stratagene).

HR was assayed by co-inoculating Δ51 ATR1 constructs with RPP1-NdA or RPP1-WsB expressing *Agrobacterium* strains, containing genomic constructs in pEarleyGate 301. Co-inoculation mixtures were infiltrated into 3–4 week old *Nicotiana tabacum* plants at OD = 0.45 per construct, as previously described [12].

### Coimmunoprecipitation assays and Western blotting

Co-immunoprecipitation was performed using 24 hour post-infiltration *N*. *benthamiana* tissue, expressing FLAG-tagged ATR1 and 3xHA-tagged RPP1. 1 g of tissue was homogenized, extracted, and processed as previously described [12], with the following changes: 10 μL of Anti-FLAG affinity gel clone M2 (Sigma) was used for immunoprecipitation, while 1:1000 FLAG M2-Peroxidase (Sigma) antibody was used for immunoblotting.

### Bacterial effector delivery and disease/HR assays

We modified the previously described EffectorDetectorVector pEDV3 [37] by exchanging the Sal1-EcoR1 fragment containing the HA tag with a linker containing Xho1-BamH1-Spe1-Flag-stop-EcoR1. This vector, pEDV3F, was used as a backbone for BamH1/Spe1 insertion of the ATR1 gain-of-recognition mutants, which were PCR amplified from the corresponding pEarleygate clones. The resulting plasmids were sequence verified and conjugated into *Pst* DC3000 and *Pf0* using triparental mating with *E*. *coli* strain PRK600.

*Pst DC3000* disease assays were performed as previously described [33,39]. Briefly, the bacteria were resuspended in 10mM MgCl_2_, adjusted to 1x10^5^ cfu/mL, and inoculated into Arabidopsis leaves with a blunt syringe. Samples were taken at day 0 and day 3, ground in 10mM MgCl_2_ with glass beads using a bead beater, diluted and plated on NYG agar containing rifampicin (50μg/ml), gentamycin (2.5μg/ml) and cycloheximide (10μg/ml).

For Arabidopsis hypersensitive response assays, *Pf0* expressing the various ATR1 alleles were grown on *Pseudomonas* agar with glycerol and the appropriate antibiotics and resuspended in 10 mM MgCl2 to 1x10^9^ cfu/ml. Half leaves of Arabidopsis were pierced and inoculated with bacterial suspension and symptoms scored at 24hpi.

### Chimeric RPP1 constructs

We constructed chimeric exchanges between RPP1-NdA and RPP1-WsB genomic constructs by overlap extension PCR. 5’ and 3’ chimeric fragments were generated by standard PCR and gel purified. Equimolar amounts of 5’ and 3’ fragments were used to self-prime an extension PCR reaction. Overlap extension reactions were carried out with 56°C annealing temperature for 15 cycles (selecting for full length chimeric templates), followed by addition of end primers and 20 additional rounds with 64°C annealing temperature. Resulting chimeric PCR product was cloned into pENTR/D-TOPO through TOPO cloning (Invitrogen), or ligated into a custom Gentamycin-resistant pENTR. Constructs were recombined into pEarleyGate 301 by LR recombination. Chimeras were tested in transient HR assays as described above.

### Homology modeling

Primary amino acid sequences for ATR1-Cala2, the RPP1-WsB NB-ARC, and LRR domains were modelled to ATR1-Emoy2, Apaf-1 NB-ARC, and Toll-like receptor 3 structures using the Phyre2 server (http://www.sbg.bio.ic.ac.uk/phyre2) [53]. Visualizations were generated using UCSF Chimera [54].

## Supporting Information

S1 Fig Co-inoculation of combined NdA-GOF and WsB-GOF mutations leads to recognition by both RPP1-NdA and RPP1-WsB. Agrobacterium inoculations were performed as in Fig. 2. WsB-GOF comprises four substitutions: V122L, S125T, Y140D, and N158K.
(TIF)

S2 Fig Multiple sequence alignment of ATR1 alleles, with NdA-GOF and WsB-GOF residues highlighted in blue and yellow respectively. The minimal recognition domain [35] is delineated in red.
(TIF)

S3 Fig Disease and avirulence phenotypes upon bacterial delivery of ATR1 GOF alleles. **(A)** Symptoms of plants inoculated as in Fig. 5 at 3 dpi. Numbers indicate proportion of replicate leaves displaying visible disease symptoms. Representative leaves are displayed with white arrows indicating leaf lesions. **(B)** Split-leaf HR phenotypes upon delivery of ATR1 alleles/mutants by *Pf0*. Plants were inoculated at OD = 1.0 and photographed at 24 hpi. Experiments were repeated 3 times with similar results.
(TIF)

S4 Fig Pairwise alignment of RPP1-NdA and RPP1-WsB with chimeric exchange regions labeled. (TIF)

S5 Fig Several chimeric RPP1 constructs express but do not recognize ATR1-Emoy2, or do not stably express. **(A)** Co-inoculation of wild-type and inactive chimeric RPP1 constructs with ATR1-Emoy2 expressing Agrobacterium. Inoculations were performed as in Fig. 2. **(B)** Weak recognition of ATR1-Cala2 NdA-GOF by WsB^598^NdA on backside of leaf. **(C)** Western blot of *N*. *tabacum* tissue collected 24 hpi expressing RPP1 constructs with 3xHA tag.
(TIF)

S6 Fig RPP1 NB-ARC and ARC1-ARC2 constructs do not co-immunoprecipitate with ATR1. FLAG-tagged ATR1 alleles were immunoprecipitated from *N*. *benthamiana* tissue extracts and probed for association with HA-tagged RPP1 subdomains by Western blot.
(TIF)

S7 Fig RPP1-NdA LRRs do not co-immunoprecipitate with recognized ATR1 variants. FLAG-tagged ATR1 alleles were immunoprecipitated from *N*. *benthamiana* tissue extracts and probed for association with HA-tagged LRRs by Western blot. RPP1-WsB LRRs were included as a positive control [12].
(TIF)

S8 Fig Homology models of the ARC2 and N-terminal LRRs of RPP1-WsB to Apaf-1 and TLR3 respectively (generated using Phyre2 server, http://www.sbg.bio.ic.ac.uk/phyre2). Polymorphic amino acid residues between RPP1-NdA and RPP1-WsB are depicted in red.
(TIF)
