# Supplementary figures and images for: Recognition and Activation Domains Contribute to Allele-Specific Responses of an Arabidopsis NLR Receptor to an Oomycete Effector Protein
Source: PLoS Pathog. 2015 Feb 11;11(2):e1004665. doi: 10.1371/journal.ppat.1004665 (PMC4335498; doi:10.1371/journal.ppat.1004665)

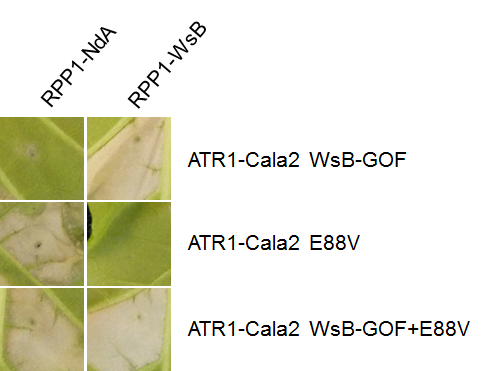

Supplement: S1 Fig — Agrobacterium inoculations were performed as in Fig. 2. WsB-GOF comprises four substitutions: V122L, S125T, Y140D, and N158K. (TIF) [file ppat.1004665.s001.tif]

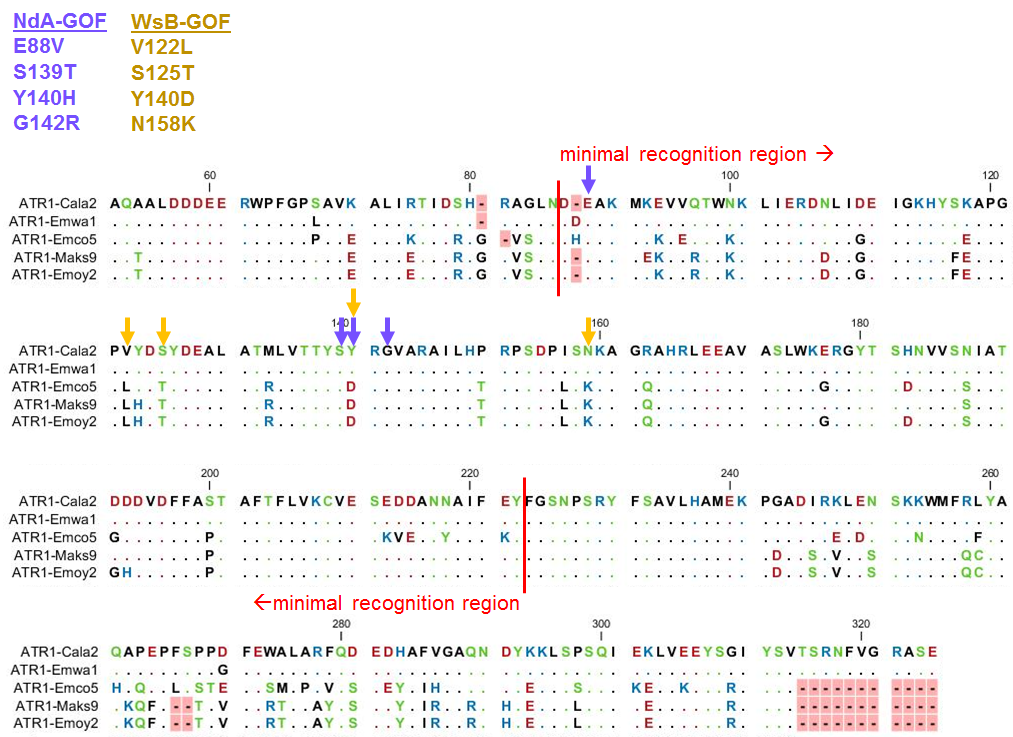

Supplement: S2 Fig — The minimal recognition domain [35] is delineated in red. (TIF) [file ppat.1004665.s002.tif]

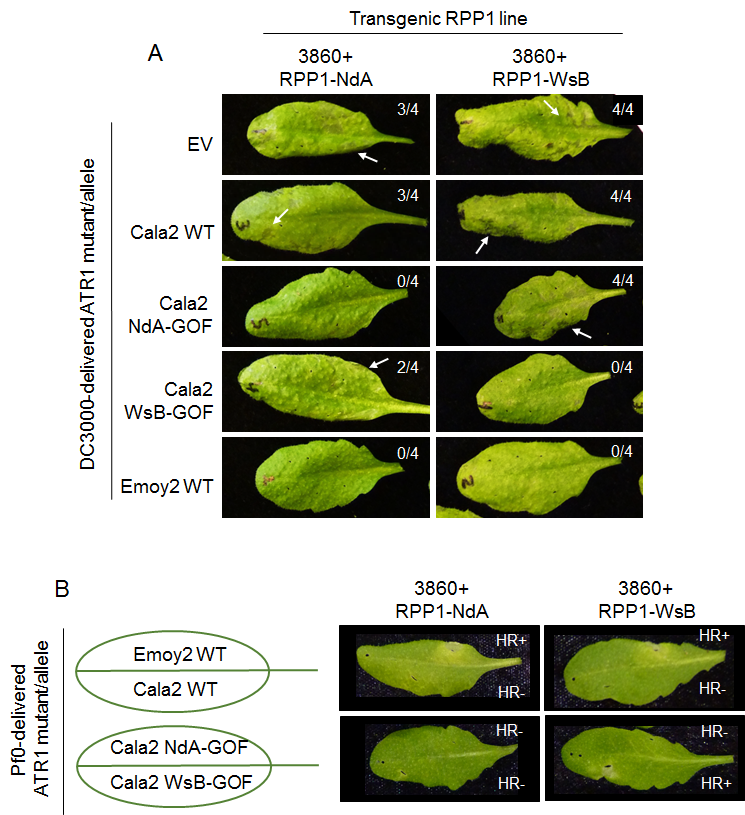

Supplement: S3 Fig — (A) Symptoms of plants inoculated as in Fig. 5 at 3 dpi. Numbers indicate proportion of replicate leaves displaying visible disease symptoms. Representative leaves are displayed with white arrows indicating leaf lesions. (B) Split-leaf HR phenotypes upon delivery of ATR1 alleles/mutants by Pf0. Plants were inoculated at OD = 1.0 and photographed at 24 hpi. Experiments were repeated 3 times with similar results. (TIF) [file ppat.1004665.s003.tif]

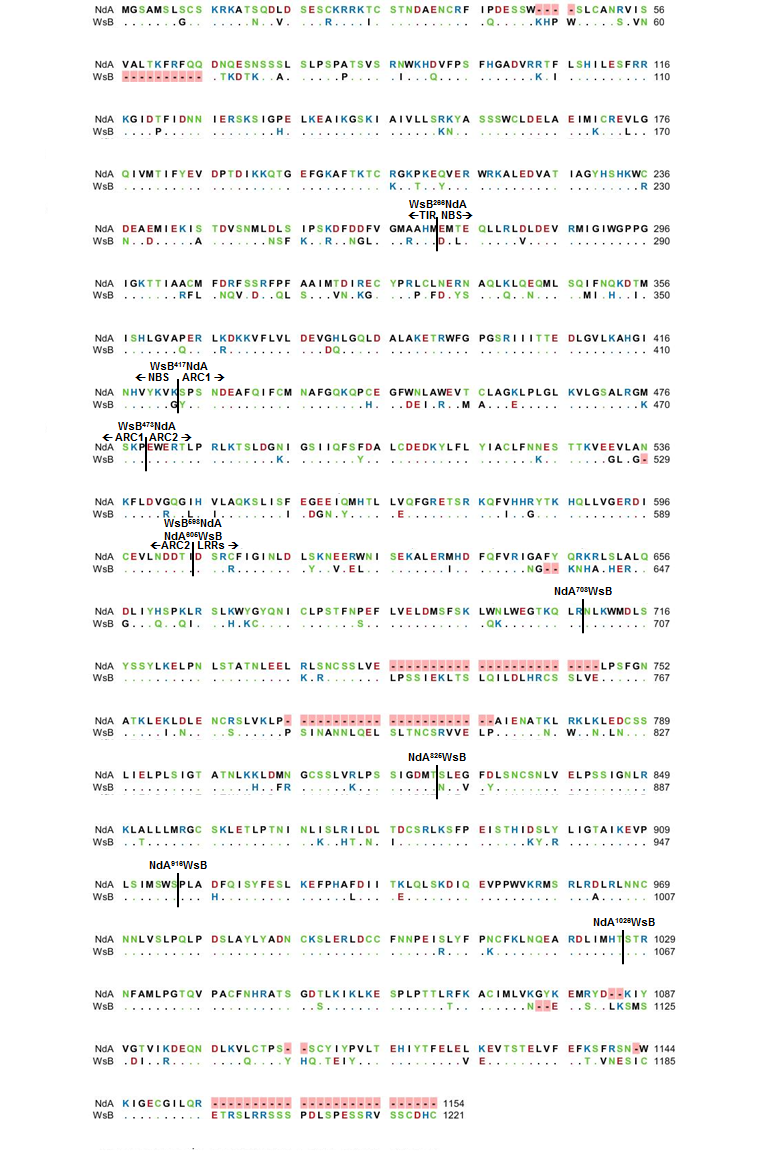

Supplement: S4 Fig — (TIF) [file ppat.1004665.s004.tif]

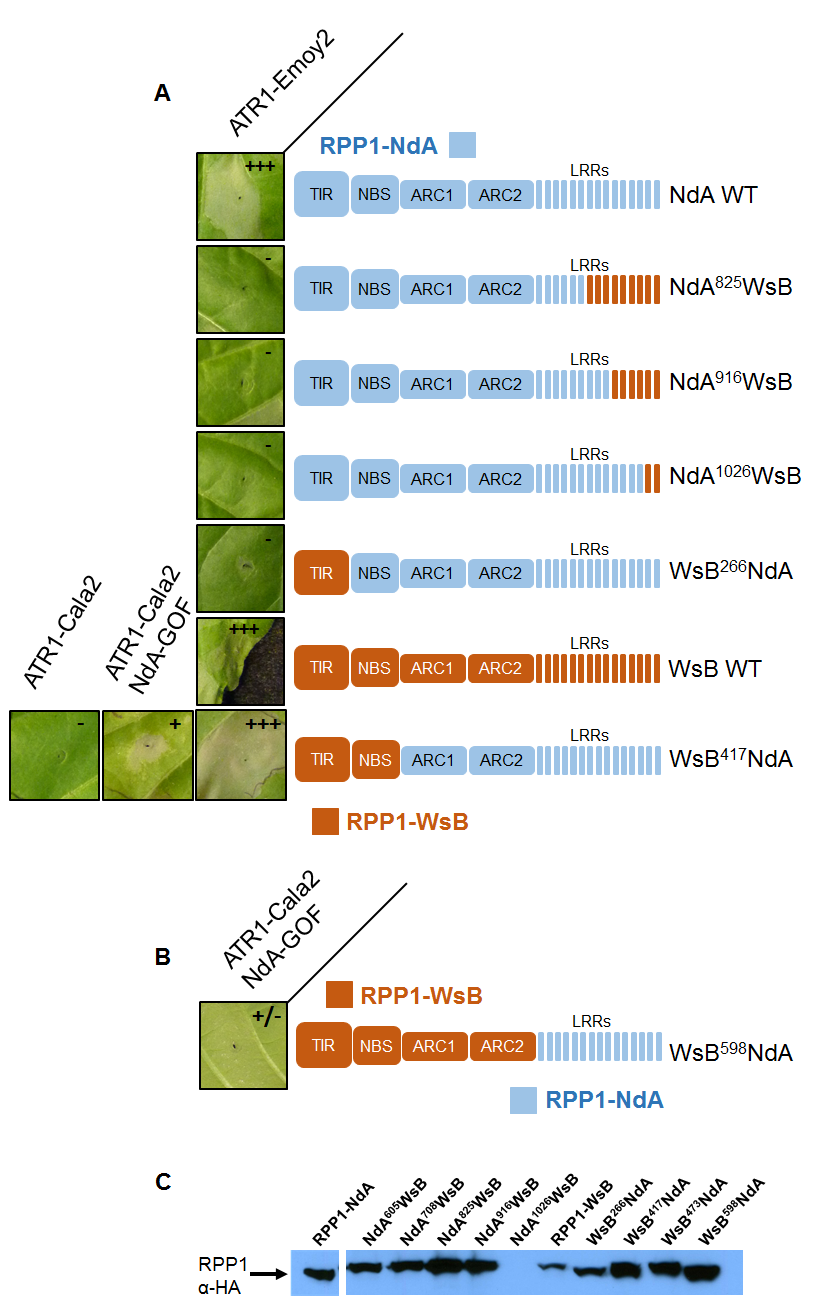

Supplement: S5 Fig — (A) Co-inoculation of wild-type and inactive chimeric RPP1 constructs with ATR1-Emoy2 expressing Agrobacterium. Inoculations were performed as in Fig. 2. (B) Weak recognition of ATR1-Cala2 NdA-GOF by WsB598NdA on backside of leaf. (C) Western blot of N. tabacum tissue collected 24 hpi expressing RPP1 constructs with 3xHA tag. (TIF) [file ppat.1004665.s005.tif]

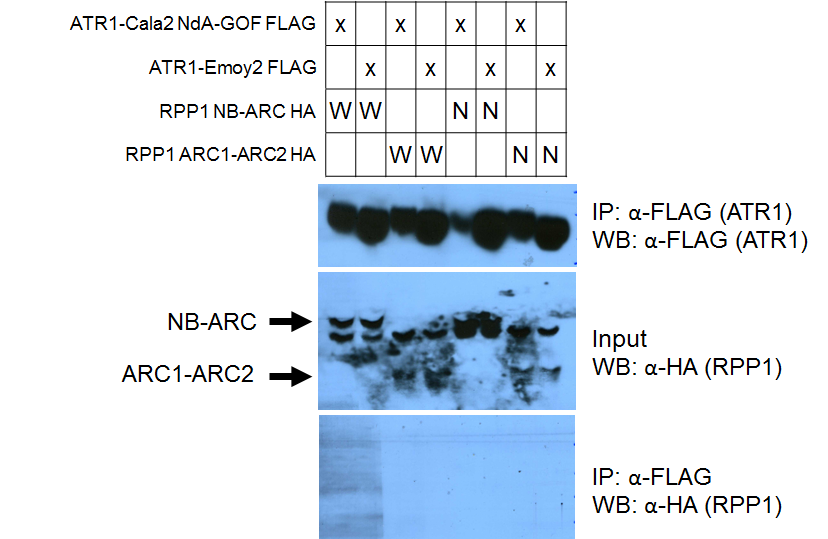

Supplement: S6 Fig — FLAG-tagged ATR1 alleles were immunoprecipitated from N. benthamiana tissue extracts and probed for association with HA-tagged RPP1 subdomains by Western blot. (TIF) [file ppat.1004665.s006.tif]

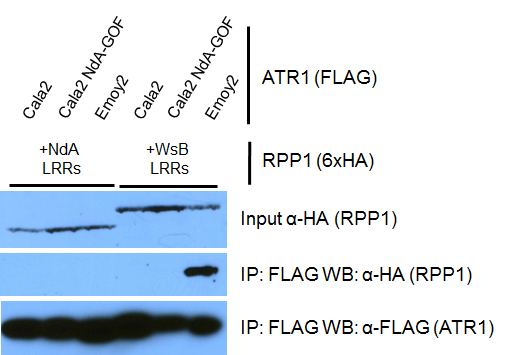

Supplement: S7 Fig — FLAG-tagged ATR1 alleles were immunoprecipitated from N. benthamiana tissue extracts and probed for association with HA-tagged LRRs by Western blot. RPP1-WsB LRRs were included as a positive control [12]. (TIF) [file ppat.1004665.s007.tif]

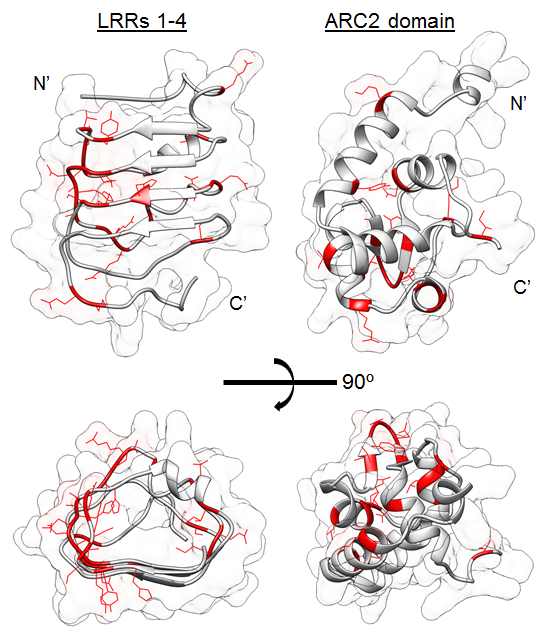

Supplement: S8 Fig — Polymorphic amino acid residues between RPP1-NdA and RPP1-WsB are depicted in red. (TIF) [file ppat.1004665.s008.tif]
